# Important Dispatch from Dr. Crouse

**Published:** 1889-06

**Authors:** J. N. Crouse

**Affiliations:** Chairman, 2231 Prairie avenve, Chicoga


### IMPORTANT DISPATCH FROM DR. CROUSE.

DEAR DOCTOR:—Did you receive the circular and By-laws recently mailed to every dentist in the United States? If not, or if you have mislaid them, will gladly send you others.

Have you read the circular? If not, why not? If you have read it, have you signed and sent By-laws, with membership fee ($10.00), as requested? If not, will you not do so at once? Also, send us history of any cases you have or know of, as described in circular.

We have already received description of many new cases, which will add valuable testimony in our favor in the coming contests.

Many have already joined the Association, but we want many more.

The amount of money required of each one is very small. The fee, however, will remain at $10.00 till a certain number is obtained; after that it will be but just, and in accord with similar organizations that those who join shall pay a larger membership fee.

We feel sure, speaking from the advice of our attorneys, Messrs. Offield and Toule, that we shall eventually be successful against all the claims of the Tooth Crown Co.

Many are asking if it will not cost more, to settle with that Company, if we *should* fail.

We answer, No, as the rates of licenses and royalty have already been established, and such rates will be held as legal in cases of past infringements and future licenses.

But we cannot fail if dentists pull together.

Will you help, or hinder by holding back?

Let us hear from you. J. N. CROUSE,

*Chairman*, 2231 Prairie avenve, Chicoga.

